# SLEEP DISTURBANCES IN RACIAL/ETHNIC DIVERSE MIDDLE-AGED AND OLDER ADULTS: FACTORS RELATED TO SLEEP DISTURBANCES

**DOI:** 10.1093/geroni/igad104.0666

**Published:** 2023-12-21

**Authors:** Alyssa Gamaldo, Angie Sardina, Jacqueline Mogle

**Affiliations:** Clemson University, Clemson, South Carolina, United States; UNC Wilmington, Wilmington, North Carolina, United States; Clemson University, Clemson, South Carolina, United States

## Abstract

Complaints of poor sleep health is a prevailing U.S. public health issue. Although complaints of sleep disturbances is common in U.S. adults, prior research has indicated rates of sleep disturbances tend to vary across racial/ethnic groups. These cross-racial/ethnic comparison approaches may be limited, however, in identifying specific and unique sociodemographic and health factors related to sleep disturbances within racial/ethnic groups. The current study explored two specific aims. The first aim was to explore racial differences in self-reported sleep disturbances. The second aim was to explore sociodemographic and health factors related to sleep disturbances within racial/ethnic groups. A sample of U.S. adults (N=521; age range=40-92) were surveyed via Qualtrics Software (December 2022). Sleep disturbances was measured by the Patient-Reported Outcomes Measurement Information System (PROMIS) questionnaire. Surveys were also collected on sociodemographic (e.g., age, monthly household income, education years, food insecurity, income changes due to COVID-19) and health (e.g., cognitive function, depressive symptoms, well-being, physical functioning, bodily pain, physical health conditions, and loneliness) factors. A univariate analysis of variance revealed non-significant racial/ethnic group differences in sleep disturbances (p > .05). However, bivariate correlations stratified by racial/ethnic groups suggested unique sociodemographic and health factors related to sleep disturbances within each racial/ethnic group. These findings contradict prior research of significant racial/ethnic group differences in sleep disturbances. Conversely, the findings support further investigating unique biopsychosocial factors associated with sleep health within racial/ethnic groups, which may provide meaningful information that may not be detected with cross-racial/ethnic comparison approaches.

